# The health benefits and practical considerations for the adoption of a Mediterranean-style dietary pattern

**DOI:** 10.1017/S0007114522002471

**Published:** 2022-10-14

**Authors:** Anne Marie Minihane, Karen Joy Murphy

**Affiliations:** 1 Norwich Medical School, University of East Anglia, Norwich, UK; 2 Clinical and Health Sciences, ARENA, University of South Australia, Adelaide, Australia

## A Mediterranean Dietary pattern (MDP) explained

The term Mediterranean diet first appeared in the late 1940s^(1)^. It is described by UNESCO as ‘a set of skills, knowledge, practices and traditions ranging from the landscape to the table, including the crops, harvesting, fishing, conservation, processing, preparation and, particularly, consumption of food’. A Mediterranean dietary pattern (MDP) varies in composition among the twenty-one countries which make up the Mediterranean region but is typically characterised by high intakes of minimally processed plant-based foods such as fruits, vegetables, nuts, seeds, legumes and whole grains. Extra virgin olive oil is the main culinary fat, with a moderate intake of dairy products and a variety of herbs and spices used as condiments, rather than salt. Fish/seafood is typically consumed two to three times per week. Red and processed meat and discretionary foods including sugar or honey-sweetened food and drinks are consumed in low amounts. Wine, and in particular red wine, is consumed in moderation and with meals. Although in many ways analogous to other global healthy plant-based dietary patterns, it is the high intakes of olive oil, nuts and red wine, which make the MDP unique. The MDP has been used as a benchmark for comparison with other dietary patterns^(2)^ and has influenced the dietary guidelines for non-Mediterranean countries like the USA, Canada and Australia.

As highlighted by Hutchins-Wiese *et al.*^(3)^, a large number of scoring systems exist to quantify adherence to a MDP, which vary in the number of components quantified, grouping of foods and approach to scoring (above or below the population median, continuous and binary scales).Such variability, although may result in a scoring system better adopted to the local context makes the integration of results from different observational studies and randomised controlled trials (RCT), and the establishment of size effects, public health messaging and clinical application, difficult.

## The evidence for the role of a MDP in cardiometabolic health

The seminal Seven Countries Study of Ancel Keys (1958–1964) and colleagues, which included a number of Mediterranean cohorts first highlighted the health benefits of this dietary pattern^(1)^. Although at times criticised for its design limitations (it has an ecological design which means that only data from average populations, not individual data is used to establish correlations), this work which included 12 763 healthy men aged 40–59 years has highlighted over the decades the strong positive and negative association between saturated fat intake and the MDP index, respectively, and CHD mortality. After 45 years follow-up, 92·9 % of the original cohort had died with a 12·9-year difference in age of CHD death between East Finland and Crete (68·0 years *v*. 80·9 years)^(4)^. A strong negative correlation between baseline MDP index and all-cause mortality at 50 years was noted^(5)^.

This association between MDP intake and CVD and all-cause mortality has been consistently reported by a number of prospective cohorts^(6)^. Including data from twenty-nine prospective studies, with 1 676 901 participants and 221 603 cases of all-cause mortality, the hazard ratio (HR) of all-cause mortality was 0·90 (95 % CI: 0·89, 0·91) for a two-point increment (ranging from 0 to 9) in MDP adherence^(7)^. Interestingly, this association was stronger in participants who lived in the Mediterranean *v*. non-Mediterranean region (HR: 0·82 compared with 0·92, respectively). Possible explanations could include: (i) high adherence to a MDP in non-Mediterranean regions differs from the pattern found with the traditional MDP followed in Mediterranean regions, where the consumption of olive oil, fish, vegetables and legumes is higher, (ii) many MDP scores use sex-specific median intake as cut-offs for each component. Therefore, because median values will differ depending on the study population, those individuals with a high adherence to a MDP in non-Mediterranean populations may be classified as poorly adherent in Mediterranean regions^(2)^, (iii) differences in bioactivate content in foods, (iv) in Mediterranean Countries a MDP score may be capturing a Mediterranean lifestyle, not fully corrected for by statistical approaches, that is, residuals and (v) MDP scores may not fully capture a high-quality diet in non-Mediterranean regions.

Although inverse associations between a MDP and CVD and overall mortality have been consistently demonstrated in observational studies, the evidence from RCT is limited and inconsistent but seems null overall^(6)^, such as in the PREDIMED study, where mortality was a secondary outcome^(8)^. However, it is likely that insufficient follow-up time and therefore small case numbers make RCT evidence inconclusive.

Most of the research on the health attributes of a MDP over the last half century has focussed on cardiometabolic health and in particular CVD and type 2 diabetes mellitus. The prospective cohort evidence for the MDP effect on incident disease or CVD outcomes^(9)^ is convincing^(6)^. A 2020 review included thirty-eight cohorts and three RCT^(10)^. Meta-analyses of the prospective cohort studies, which compared the highest *v*. lowest MDP adherence, revealed an inverse association with total CVD mortality (relative risk (RR): 0·79; 95 % CI: 0·77, 0·82), CHD incidence (RR: 0·73; 95 % CI: 0·62, 0·86), CHD mortality (RR: 0·83; 95 % CI: 0·75, 0·92), stroke incidence (RR: 0·80; 95 % CI: 0·71, 0·90), stroke mortality (RR: 0·87; 95 % CI: 0·80, 0·96) and myocardial incidence (RR: 0·73; 95 % CI: 0·61, 0·88)^(10)^. For the three RCT (PREDIMED, Indo-Mediterranean diet heart study and the Lyon diet heart study), a 38 % reduction in CVD incidence (RR: 0·62; 95 % CI: 0·50, 0·78) and a 35 % reduction in total myocardial infarction incidence (RR: 0·65; 95 % CI: 0·49, 0·88) were observed^(10)^.

## A MDP and brain health

More recently, the MDP or modifications of the MDP such as the MIND diet have been shown to improve neurocognitive function, reduce the risk of dementia and Alzheimer’s disease and reduce brain atrophy and neuropathology^(11,12)^. Given there is no current long-term pharmacological options for the treatment of cognitive decline or dementia, there is a great need for such diet and other lifestyle strategies.

Although the long-term effects of a MDP on cognition and overall mental well-being have been consistently described, the short-term effects of the MDP on cognitive performance, mood and anxiety have not been as widely investigated. A recent systematic review, whilst limited to three articles and a conference proceeding have suggested that in the short-term, up to 10 days, a MDP can improve cognitive function and mood. Specifically, improvements in attention, alertness and contentment were consistently reported^(13)^.

Globally depression is a common illness, and according to the World Health Organisation around 3·8 % of the population are estimated to be affected. Meta-analyses of observational studies and RCT have shown that healthy diets such as the DASH diet (Dietary Approaches to Stop Hypertension) as well as the MDP are associated with a lower risk of depression^(14–16)^. Interestingly, most of the research around depression is conducted in women and older adults; however, men are less likely to seek help for depression. Bayes and colleagues conducting a 12-week RCT, the ‘AMMEND’ study, to examine the effect of a MDP on symptoms of depression in young men aged 18–25 years. The primary outcome is the Becks depression inventory score^(17)^. The postprandial/acute effects of the MDP on depression, mood and quality of life are largely unknown; however, there are several studies in progress, exploring these outcome measures.

The relationship between adherence to a MDP and health-related quality of life is also unclear, particularly in vulnerable older adults. A cross-sectional analysis by Cordwell and colleagues explored the associations in two independent cohorts of overweight and obese middle-aged to older adults with and without type 2 diabetes mellitus. In the adjusted model, using pooled data from both study cohorts, adherence to a MDP was associated with the general health subscale of health-related quality of life. Similar findings were also observed in the type 2 diabetes mellitus cohort. However, no additional significant associations between adherence to a MDP and health-related quality of life subscales were observed^(18)^.

## A MDP, inflammation and other chronic conditions

Inflammation is a common underlying cause of many chronic diseases. Adherence to a MDP is associated with reduced inflammation and a lower dietary inflammatory index score. A study from Iran investigated the potential of a modiﬁed MDP to improve dietary inflammatory index scores, disability and fatigue severity, in relapsing-remitting multiple sclerosis compared with a traditional Iranian diet, in 180 patients. Adherence to a MDP for 6 months improved dietary inflammatory status and fatigue severity relative to the traditional Iranian diet^(19)^. As reviewed by Clark and colleagues, the protective effects of the MDP against inflammation may be in part mediated through favourable changes to the gut microbiota composition and activity^(20)^.

Emerging evidence is focussing on the relationship between consumption of a MDP and many other chronic diseases^(21)^, including non-alcoholic fatty liver disease (NAFLD)^(22)^, cancers and colorectal cancer^(23)^, multiple sclerosis^(19)^, as well as positive roles in athletic performance^(24)^ and weight management in pregnancy^(25)^.

Previous RCTs investigated the effects of MDP on features of the NAFLD phenotype like liver enzymes, but their results are contradictory. A study by Sangouni and colleagues aimed to systematically review and meta-analyse available RCTs. A total of ten RCTs (*n* 705 participants) evaluating the effect of a MDP on liver enzymes including aspartate aminotransferase, alanine transaminase and γ-glutamyltransferase were included. The MDP significantly reduced aspartate aminotransferase and γ-glutamyltransferase but not alanine transaminase^(26)^. Mayr and colleagues explored the multidisciplinary clinicians’ perspectives on whether the MDP is recommended in the routine management of NAFLD and barriers and facilitators to its implementation in a multi-ethnic setting. Clinicians described lifestyle modification as the first line of treatment and diet was recognised as everyone’s role whilst dietitians provide specific individualised dietary care. Several barriers were noted including cultural and socio-economic backgrounds but also time and resources to support behaviour change. Further innovations to service delivery could better support and empower patients to change dietary behaviour long term^(22)^.

Cancer is the leading cause of death worldwide^(27)^ with colorectal cancer as one of the most commonly diagnosed cancers. Olive oil which is part of a MDP contains numerous phenolic compounds that mitigate free radical-induced cell damage and inflammation. The recent review by Sain and colleagues focussed on the protective role of olive oil and its phenolic compounds against colorectal cancer as well as exploring the cellular and molecular signalling mechanisms. However, further research is needed to establish whether these compounds have a preventive or therapeutic role in colorectal cancer^(23)^.

The health benefits of olive oil have been studied independently from the MDP. In a rodent model of arterial hypertension, extra virgin olive oil demonstrated vasodilator, antihypertensive, antioxidant as well as antifibrotic and hypertrophic properties^(28)^.

## A MDP beyond disease risk reduction

The exploration of the benefits of the MDP has extended beyond chronic disease risk reduction to an examination of its effect on maternal and infant health^(25)^, adolescent well-being^(29)^ and athletic performance^(24)^.

Gestational weight gain and postpartum weight retention are considered critical determinants of nutritional status. Radwan and colleagues^(25)^, using data from the Mother-Infant Study Cohort (MISC), explored adherence to the MDP during pregnancy and relationships between gestational weight gain and postpartum weight retention. The findings of this study showed that adherence to the MDP may reduce gestational weight gain and postpartum weight retention and highlights the importance of promoting the MDP for better health of the mother and infant.

Two studies explored levels of adherence to an MDP in adolescents^(29,30)^. Jimenez-Boraita et al^(29)^ showed being female and having higher levels of physical activity (PA) were found to be predictive factors of adherence to the MDP. In addition, maximum oxygen consumption, the presence of environments favourable towards PA engagement and higher self-esteem were also predictive in females, whilst better academic performance and more ‘nightly sleep’ were additional predictors in males. In Lebanon, a cross-sectional survey with 798 adolescents aged 11–18 years showed lower adherence to the MDP was correlated with skipping meals (*P* = 0·001), whilst adolescents who were engaged in a high level of PA, those who consumed more meals with their families and those who benefited from better physical well-being had better diet quality^(30)^.

Appropriate nutrition plays a major role in sport. In a narrative review, Griffiths and colleagues suggested from preliminary evidence that consumption of a MDP, and its individual components, could play a role in optimising certain aspects of health and performance in competitive athletes^(24)^.

The relationship between adherence to the MDP, PA, sedentary behaviour and physical fitness levels was meta-analysed using thirty-nine studies with 565 421 children and adolescents (mean age, 12·4 years). Overall, the MDP had a weak-to-moderate positive relationship with PA, cardiorespiratory fitness and muscular fitness, and a small-to-moderate negative relationship with sedentary behaviour and speed agility; however, there was a high level of heterogeneity in all the models. Except for PA, results did not remain significant after controlling for sex and age (children or adolescents)^(31)^.

## MDP adoption approaches

Much of the MDP-related research has adopted a discovery science approach. Little research focus has been given to date on how to effectively change and sustain population eating behaviours so that it is more MDP adherent, particularly in non-Mediterranean regions, taking into account the social, cultural, environmental, geographical and economic differences^(32,33)^. Current trials that are in progress include MedWalk - a Mediterranean diet intervention combined with walking (Trial Registration, ACTRN12620000978965) which includes strategies like motivational interviewing cognitive behavioural therapy to assist participants in making healthy behaviour change to ensure changes to diet and lifestyle are feasible to adopt long term. Similarly, MedEx is a recently completed RCT that has examined the feasibility of adopting a MDP and a MDP plus PA intervention in UK adults at risk of age-related cognitive decline. MedEx utilised strategies including self-selected dietary targets, group sessions, digital support and food deliveries to help individuals make and sustain healthy lifestyle choices^(34)^. The lack of research evidence and potential benefits of a combined MDP and PA was reviewed by Hershey and colleagues^(35)^. The authors also prospectively assessed the impact of MDP and PA on all-cause mortality in the Seguimiento Universidad de Navarra (SUN) cohort, followed by both multiplicative and additive interaction analyses. The SUN cohort is a prospective open-recruitment study in Spanish university graduates with a baseline and biennial follow-up questionnaires. Spanish university graduates have participated since 1999, and the cohort now has over 22 500 participants. Hershey et al reported those participants with higher MDP adherence had higher PA compared with those with lower PA. Similarly, lower adherence to the MDP and low level of PA was associated with an increased risk of mortality compared to the highest MD adherence quartile and the highest PA category.

TEAM-MED, The Trial to Encourage Adoption and Maintenance of a MEditerranean Diet, was a 12-month pilot parallel group RCT involving individuals aged ≥ 40 years, with low MDP adherence, who were overweight and had an estimated CVD risk ≥ 20 % over 10 years^(32)^. This study aimed to explore methods of increasing MDP adoption in a non-Mediterranean population at high risk of CVD, including assessing the feasibility of a developed peer support intervention. This pilot study has demonstrated that a non-Mediterranean adult population at high CVD risk can make dietary behaviour change over a 12-month period towards a MDP. The study also highlights the feasibility of a peer support intervention to encourage MDP behaviour change amongst this population group and will inform a definitive trial^(33)^.

Rodrigues and colleagues^(36)^ sought to develop a Portuguese Mediterranean diet wheel. Three main steps were followed: (1) establishment of the most relevant Mediterranean diet and lifestyle principles to improve this pattern among the Portuguese population; (2) converting those principles into a captivating and easy-to-understand tool and (3) obtaining experts’ opinion. This newly developed Mediterranean food guide is an educational tool that will support health and education professionals to promote healthy food choices.

Furthermore, it is widely accepted that a traditional MDP is reflective of a lifestyle, which includes conviviality and social interaction, regular PA and adequate sleep and locally sourced food. Therefore, a somewhat unanswered question is the benefits of a MDP in isolation without these associated contextual health-enhancing elements of a traditional Mediterranean lifestyle. This edition has provided insights into the additive/synergistic effects of a MDP with PA^(3,20,35)^ and also the association between various lifestyle/behaviour factors and MDP adherence particularly in adolescents^(29–31)^. Knowledge of such inter-dependence is hugely important for the design and delivery of future public health^(36)^ and clinic practice^(22)^ guidelines.

### Conclusion

There is strong and consistent evidence showing that a MDP promotes well-being and reduces chronic disease. Research to date has focussed mainly on cardiometabolic health and, to a lesser degree brain health, with further long-term prospective studies and well-conducted RCT on other outcomes needed. It is currently assumed that the often large beneficial effect sizes are due to the additive and even synergistic effects of individual foods and bioactives. It may be that certain MDP components or their composition, for example, types of fruit and vegetables, are more beneficial depending on the health outcome of interest, which remains to be fully established.

In addition to the fundamental and efficacy research, there is a great need to consider sustainable food systems which provide access to an affordable MDP (or other similar healthy plant-based diets) and to community eating behaviour and health professional training approaches which provide the opportunity and motivation for MDP adoption.
